# Discovery of a Novel Inhibitor of the Protein Tyrosine Phosphatase Shp2

**DOI:** 10.1038/srep17626

**Published:** 2015-12-02

**Authors:** Chuan Chen, Mengmeng Cao, Siyu Zhu, Cuicui Wang, Fan Liang, Leilei Yan, Duqiang Luo

**Affiliations:** 1 College of Life Science, Key Laboratory of Medicinal Chemistry and Molecular Diagnosis of the Ministry of Education, Hebei University, Baoding, Hebei 071002, P.R. China

## Abstract

Shp2 is a ubiquitously expressed protein tyrosine phosphatase (PTP) related to adult acute myelogenous leukemia and human solid tumors. In this report, we describe identification of a potent Shp2 inhibitor, Fumosorinone (Fumos) from entomogenous fungi, which shows selective inhibition of Shp2 over other tested PTPs. Using a surface plasmon resonance analysis, we further confirmed the physical interaction between Shp2 and Fumos. Fumos inhibits Shp2-dependent activation of the Ras/ERK signal pathway downstream of EGFR, and interrupts EGF-induced Gab1-Shp2 association. As expected, Fumos shows little effects on the Shp2-independent ERK1/2 activation induced by PMA or oncogenic Ras. Furthermore, Fumos down-regulates Src activation, inhibits phosphorylation of Paxillin and prevents tumor cell invasion. These results suggest that Fumos can inhibit Shp2-dependent cell signaling in human cells and has a potential for treatment of Shp2-associated diseases.

The antagonizing effect of protein-tyrosine kinases (PTKs) and protein-tyrosine phosphatases (PTPs) regulates cellular processes such as proliferation, survival, differentiation, migration and apoptosis^1^. The role of PTKs in the development of human diseases has been a research focus for years. Recently, accumulating evidence indicates significant roles of some PTPs, such as Shp2, PTP1B, CDC25 and PRL3 in the development of some human diseases, cancer in particular^2^,^3^. Shp2 is the first confirmed bona fide proto-oncogene among the PTP superfamily. Shp2 is encoded by PTPN11 and contains two tandem N-terminal SRC homology 2 (SH2) domains, N-SH2 and C-SH2, a catalytic PTP domain, and a C-terminal tail with tyrosyl phosphorylation sites and a proline-rich motif. The N-terminal SH2 domain binds to the PTP domain, resulting in Shp2 auto-inhibition. This auto-inhibition can be relieved by the association of Shp2 SH2 domains with docking proteins phosphorylated at tyrosine sites, and this activation process is stimulated by growth factors or cytokines^1^.

Shp2 plays an important role in various cell signaling events for metabolism, proliferation, differentiation, migration and survival. Depending on cell types or receptors, Shp2 regulates the activity of Ras-ERK (extracellular signal-related kinase), PI3K-Akt, NFAT (nuclear factor of activated T cells) and the NF-κB (nuclear factor kappa B) pathways^4^. Previous studies show that Shp2 is required for full activation of the mitogen-activated protein kinase (MAPK)/ERK pathway downstream of most receptors such as Met, fibroblast growth factor (FGF), epidermal growth factor (EGF) and insulin receptor^5^,^6^.

Gain-of-function mutations of Shp2 that disrupt the auto-inhibition were reported in Noonan Syndrome (NS), LEOPARD syndrome (LS) and juvenile myelomonocytic leukemia, but Shp2 mutations occur at low frequency in solid tumors^7^. Upregulation of Shp2 expression has been reported in other human cancers, including breast cancer, liver cancer, gastric cancer, oral cancer, non–small cell lung cancer and thyroid cancer^8^,^9^,^10^,^11^,^12^,^13^,^14^. This makes Shp2 an excellent target for the development of therapeutic drugs. However, some reports found that Shp2 decreases in some types of tumors and the loss of cytoplasmic Shp2 expression is associated with increased growth and cancer progression^15^,^16^,^17^. Organ-specific PTPN11 deletion showed that shp2 acts as a tumor suppressor in cartilage and hepatocellular carcinoma^7^,^18^. Based on these controversial reports, Wang proposed that Shp2 plays dual roles in liver cancer, either suppressing or promoting the development of hepatocellular carcinoma^13^.

Many Shp2 inhibitors have been identified so far, but majority of the reported inhibitors shows low selectivity for Shp2 over other PTPs, presumably due to the highly conserved active pocket shared by all PTPs^19^. However, several characterized Shp2 inhibitors, such as PHPS (NSC-87877) and NSC-117199, exhibit specificity toward Shp2 over other PTPs^20^,^21^. Moreover, phenylhydrazonopyrazolone sulfonate (PHPS1) has been identified as a cell membrane-permeable inhibitor specific to Shp2 compared to closely related PTPs such as Shp1 and PTP1B^22^. Other Shp2-specific inhibitors, such as DCA, Cryptotanshinone, II-B08 and #220–324, were also identified and characterized^23^,^24^,^25^,^26^,^27^.

In recent years, there has been a growing interest in PTP inhibitors from natural products. To date, very few PTPs inhibitors have been isolated from microorganisms, in particular insect pathogenic fungi. Therefore, insect pathogenic fungi have been considered as an untapped source of small molecules PTP inhibitors. In our previous study, we have isolated a new compound, named Fumosorinone (Fumos) from insect pathogenic fungi^28^, which was found to improve insulin resistance in type 2 diabetes^29^. In this study, we identified Fumos as a potent Shp2 inhibitor. Fumos shows selective inhibition on Shp2 over other PTPs. Fumos also inhibits tumor cell proliferation, attenuates Shp2-dependent Ras-ERK1/2 activation induced by EGF, and reduces invasion of HeLa cells and MDA-MB-231 cells by down-regulating Src signaling pathway.

## Results

### Identification of Fumos as a Shp2 Inhibitor

**Figure 1:**
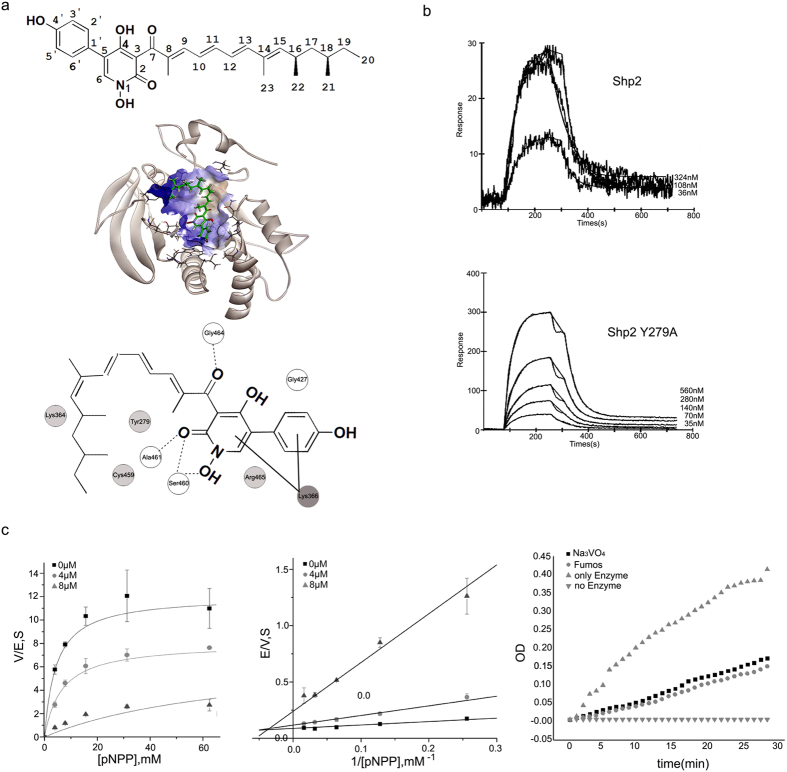
Molecular docking and kinetic analysis of Fumos binding to the Shp2. (a) Up:the structure of Fumos. Middle: Molecular docking by Discovery Studio. Fumos is showed in stick model, carbon atoms are colored in green. Down: Docking Scheme The hydrogen bonds form between Fumos and the protein via Ser460, Gly464, Ala461and Gly427 (white balls). The dotted lines reprsent hydrogen bonds. The hydrophobic bonds form between Fumos and the protein via Tyr279, Cys459, Lys364 and Arg465 (light grey balls). Lys366(dark grey balls) interacts with Fumos via static electricity. (**b**) Surface plasmon resonance (SPR) analysis of Shp2 and Shp2 Y279A binding to Fumos. Fumos was immobilized to a biosensor chip and analyzed for binding of Shp2 and Shp2 Y279A. Tracings of a typical experiment showed the binding of increasing amounts of Shp2 and Shp2 Y279A to Fumos. (**c**) Kinetic analysis of Shp2 inhibition by Fumos. Enzyme kinetics of Shp2 varying the concentrations of substrate (pNPP) and Fumos. Concentrations of Fumos were selected at 0 μM (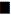), 4 μM (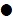) and 8 μM (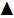). Left: Michaelis-Menten model Middle: the Lineweaver-Burk plot Right: Recombinant Shp2 was preincubated for 30 min with Fumos and Sodium Orthovanadate at the respective IC_50_ value in the absence of substrate, and then diluted and assayed for enzymatic activity. Bars represent mean ± S.E.M. values from three experiments.

To identify novel small molecule inhibitors of Shp2, we screened a diverse collection of the second metabolites of entomogenous fungi using an *in vitro* PTP assay with a His-tagged human Shp2 protein. As a result, a novel Shp2 inhibitor, Fumos, a 2-pyridone alkaloid was identified (Fig. 1a). To further assess its PTP specificity, the inhibitory effect of Fumos on the PTP domain of Shp2 and other human PTPs was examined *in vitro*. As shown in Table 1, Fumos inhibited the PTP activity of Shp2 with an IC_50_ of 6.31 μM, but showed very little inhibition toward other human PTPs (HePTP, Lyp, STEP, PTPH1, PTPRA, Cdc25b and MEG2). Although Fumos exhibited some inhibitory effects on PTP1B, TCPTP, and Shp1, whose structures are closely related to Shp2, the values of IC_50_ are much higher than that of Shp2, indicating its particular specificity toward Shp2 (Table 1). Compared with the PTP domain of Shp2, the full-length Shp2 (Shp2 FL) is also sensitive to Fumos, but the IC_50_ is relatively higher (26.52 μM, Table 1), suggesting that the SH2 domains of Shp2 may interfere with the inhibitory activity of Fumos. As a commonly used inhibitor for PTPs, sodium orthovanadate inhibited the enzyme activity of the Shp2 PTP domain with an IC_50_ of 620 μM in our PTP assay. Thus, compared to, Sodium orthovanadate Fumos can inhibit the enzyme activity of Shp2 with higher efficiency. Taken together, our results indicate that Fumos specifically inhibits Shp2 enzyme activity.

### Molecular docking of Fumos with Shp2

Next, we assessed the Fumos binding sites in Shp2 using computer docking and structural analysis. The PTPase signature motif, also known as the PTP loop, is made of the following residues (I/V)HCXAGXGR(S/T)G^30^. Results of computer docking showed that Fumos forms four hydrogen bonds with the PTP loop of Shp2 (Gly464, Arg465, Ala461, Ser460), as well as two hydrophobic interactions with Cys459 and Arg465 in the PTP loop. Additionally, computer docking modeling suggests that Fumos interacts with Shp2 in the vincinity of the active site (Fig. 1a). Binding of phosphopeptides to the PTP loop promotes a major conformational change in the catalytic site surface that is defined as WPD loop^30^. Fumos is predicted to form a hydrogen bond with Gly427 in the WPD loop and a hydrophobic bond with Tyr279 in phosphotyrosine recognition loop. To evaluate this molecular model, we constructed a Shp2-Y279A mutant that contains change in the Tyr-279 residue predicted to interact with Fumos. Interestingly, Shp2-Y279A did not show any enzyme activity, suggesting an indispensable function of Y279 for the enzyme activity of Shp2. The specific interactions observed with Shp2-Y279A and Shp2-WT with Fumos were subsequently confirmed by surface plasmon resonance(SPR) analysis using purified proteins. The results revealed that Fumos binds to Shp2-WT with a K_a_ = 7.06 × 10^5^ M^−1^ S^−1^, K_d_ = 2.85 × 10^−2^ S^−1^, K_D_ = 40 nM (Fig. 1b). SPR analysis revealed that Fumos binds to Shp2-Y279A with K_a_ = 5.06 × 10^4^ M^−1^ S^−1^, K_d_ = 1.86 × 10^−2^ S^−1^, K_D_ = 368 nM, while Fumos binds to Shp2-WT with K_D_ = 40 nM (Fig. 1b). Thus, Shp2-Y279A exhibits nine-fold less affinity to Fumos, indicating the critical role of Tyr279 in the physical interaction between Fumos and Shp2. We also compared the predicted Fumos binding sites in Shp2 with other PTPs and found that Tyr279, Lys366, Gly427 and Lys364 are not conserved in other PTPs. This difference further suggests that Fumos is likely a Shp2-specific inhibitor.

### Fumos is a non-competitive inhibitor of Shp2

In order to determine the nature of the inhibition, the phosphtase activity of Shp2 was assessed in the presence of increasing concentrations of Fumos. Lineweaver-Burk analysis suggested that Fumos decreased the Vmax without affecting Km(Fig. 1c). From the fitting we obtained a Km of 6.75 ± 0.84 mM and a series of varying Vmax of 12.05 ± 0.60 S^−1^, 7.63 ± 0.42 S^−1^ and 2.73 ± 0.41 S^−1^ corresponding to the varied fumosorinone concentrations of 0, 4 and 8 μM, while the double-reciprocal plot shows all the straight lines focus in the second quadrant and demonstrate a pattern of intersection close to the X-axis. This suggests that fumosorinone is a classic noncompetitive inhibitor for Shp2. Since irreversible inhibitors generally show similar variation trend with non-competitive inhibitors in Lineweaver-Burk plot, next we investigated if the inhibition of Shp2 by Fumos is reversible. Unlike an irreversible inhibitor, a reversible inhibitor can dissociate from the enzyme. Non-competitive inhibition can be completely reversed by adding more substrates so that it reaches a much higher concentration than that of the inhibitor^31^,^32^. After incubation of the mixture containing Fumos and Shp2, we added 40-fold of normal amount of substrates. Sodium orthovanadate, a commonly used competitive inhibitor for PTPs, was used as a control. As shown in Fig. 1c, the enzyme activity of Shp2 was recovered after addition of large amount of substrates. Reactions with either Fumos or sodium orthovanadate exhibited similar recovery. These data indicate that the inhibition of Shp2 by Fumos follows the Michaelis–Menten equation for a non-competitive inhibitor.

### Fumos inhibits EGF-induced ERK1/2 activation

**Figure 2:**
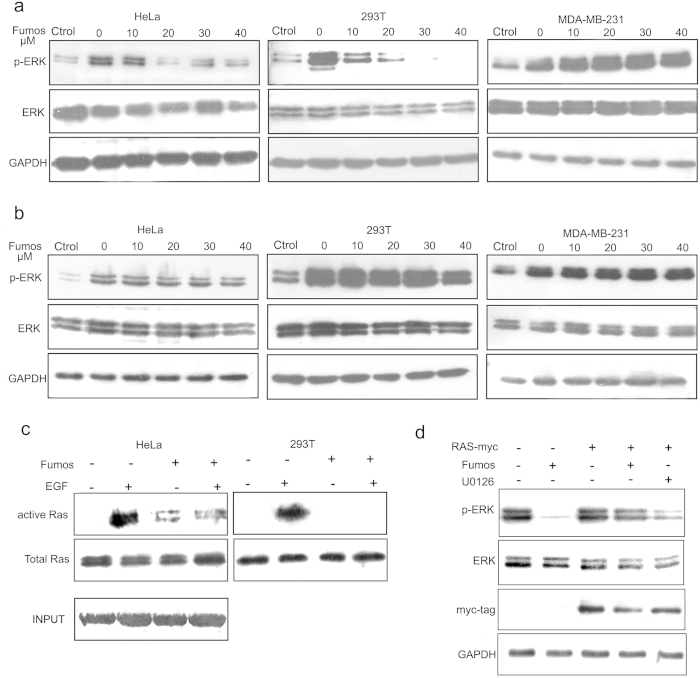
Fumos inhibits Shp2-dependent signaling pathways. (**a**) Fumos inhibits EGF-induced ERK1/2 activation. Serum starved cells were pretreaed with various concentrations (0, 10, 20, 30, 40 μM) of Fumos for 24 h and then stimulated with EGF (20 ng/ml, 5 min). The control treatment is not induced by EGF. (**b**) Fumos doesn’t inhibit PMA-induced ERK1/2 activation. Cells were pretreated with increasing concentrations of Fumos for 24 h, and then stimulated with 100 nM PMA for 5 min. DMSO treatment as control. ERK1/2 activation was analyzed by Western-Blot with antibodies to phosphorylated ERK1/2 (pERK) or ERK1/2. GAPDH is used as a loading control for Western Blot. The control treatment is not induced by PMA. (**c**) Fumos inhibits EGF-induced Ras activation. Serum-starved cells were treated with Fumos(20 μM) and EGF as indicated. Cell lysates (0.5 mg of protein/each) were incubated with GST-RBD-agarose to pull down active-Ras-GTP, which was visualized by immunoblotting with an anti-Ras antibody. (**d**) Fumos doesn’t inhibit RasV12-induced ERK1/2 activation. HEK 293T cells were transiently transfected with a pCMV vector for RasV12 and empty vector, and treated with Fumos(20 μM) or U0126(10 μM) for 24 h, respectively. Cell lysates were immunoblotted with Myc-tag indicating the expression of RasV12. Total cell lysates were immunoblotted with antibodies for p-ERK1/2 and ERK1/2.

Shp2 is required for full activation of EGF-induced ERK1/2^33^. To determine whether Fumos inhibits Shp2-dependent ERK1/2 activation downstream of EGFR, we used three human cell lines (HeLa, MDA-MB-231 and HEK293T) to examine the the phosphorylation/activation of ERK1/2. HeLa and HEK293T cell lines contain wild type Ras, whereas MDA-MB-231 cells have oncogenic mutation in K-Ras (G13D). Serum starved cells were pretreated with various concentrations (0, 10, 20, 30, 40 μM) of Fumos for 24 h and subsequently stimulated with EGF (20 ng/ml, 5 min). As shown in Fig. 2a, ERK1/2 phosphorylation was increased after treatment with EGF in the absence of Fumos. In the presence of Fumos, however, ERK1/2 phosphorylation was drastically decreased in HeLa and HEK293T cell lines. Suprisingly, the decrease was not observed in MDA-MB-231 cell line. The results suggest that Fumos inhibits EGF-stimulated ERK1/2 activation in a dose-dependent manner in HeLa and HEK293T cell lines that contain wild type Ras.

### Fumos does not affect PMA-induced ERK1/2 Activation

Previous studies have shown that phorbol 12-myristate 13-acetate (PMA)-induced ERK1/2 activation is independent of Shp2^20^. Instead, the PMA-induced activation is mediated by protein kinase C, which activates the ERK1/2 MAP kinase cascade by directly phosphorylating Raf-1^34^. Accordingly, we found that Fumos did not inhibit PMA-induced ERK1/2 phosphorylation even at the highest concentration tested (Fig. 2b). This result suggests that Fumos may not inhibit ERK directly and it likely functions upstream of Raf-1.

### Fumos inhibits EGF-stimulated Ras activation

The PTP activity of Shp2 is required for full activation of Ras, and thereby Shp2 functions upstream of Ras^35^. To determine whether Fumos inhibits Ras activation, GST-RBD (Ras-binding domain) pull-down assay was used to analyze EGF-induced Ras activation in HeLa and HEK293T cells. Active Ras binds specifically to the RBD of Raf1, leading to its activation^36^. Therefore, GST-RBD pull-down assay can be used as to specifically isolate the active form of Ras. As shown in Fig. 2c, the result showed that endogenous Ras activation was inhibited by Fumos in HeLa and HEK293T cells.

Our data indicate that Fumos does not inhibit EGF-stimulated ERK1/2 activation in MDA-MB-231 cell line (Fig. 2a). One possible explanation is that Shp2 functions upstream of Ras in the Ras-ERK1/2 MAP kinase pathway and does not inhibit ERK1/2 phosphorylation induced by mutation K-Ras (G13D) in MDA-MB-231 cells. To test, we determined whether Fumos inhibits EGF-stimulated ERK1/2 activation in other cell lines expressing mutant Ras. We transiently expressed mutant Ras-V12, a constitutively active form of Ras, in the HEK293T cells and analyzed the effect of Fumos on the activation of ERK1/2. Interestingly, Fumos did not inhibit ERK1/2 activation in the HEK293T cells expressing H-RasV12 (Fig. 2d). As a positive control, a selective inhibitor of MAP kinase U1206 blocked ERK1/2 phosphorylation. These findings indicate that Fumos inhibits EGF-induced ERK1/2 activation. Because Fumos acts upstream of Ras, it is incompetent in cells with constitutively active Ras. Therefore, our data support the conclusion that Fumos inhibits Ras/MAP kinase signaling and acts upstream of Ras.

### Fumos affects Gab1-Shp2 association

**Figure 3:**
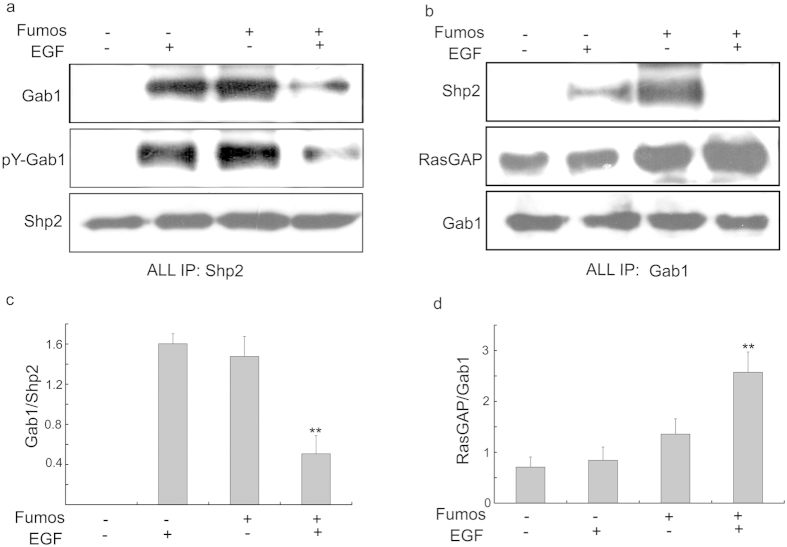
Fumos inhibits Gab1-Shp2 association. HEK293T cells were serum-starved for 18 h, preincubated with or without Fumos (20 μM 24 h), and then treated with EGF (20 ng/ml, 5 min). (**a**) Shp2 was immunoprecipitated from cell lysate supernatants. (**b**) Gab1 was immunoprecipitated from cell lysate supernatants. Immunoprecipitates were analyzed by immunoblotting with antibodies to Gab1, RasGAP, pY and Shp2. (**c**) Densitometric analysis of p-Gab1/Shp2 ratio in the Western blots. **p  = 0.002 < 0.01/F = 37 vs EGF. (**d**) Densitometric analysis of RasGAP/Gab1 ratio in the Western blots. **p < 0.01/F = 30 vs EGF, one-way ANOVA followed by Tukey’s test. Bars represent mean ± S.E.M. values from three experiments.

Shp2 can directly bind the docking protein Gab1or Gab2, which leads to Ras/ERK activation in EGF-stimulated cells^37^. Because HEK293T cells express Gab1 but not Gab2^20^, it is a proper cell line to study the Gab1-Shp2 association and exclude the interference from Gab2. We analyzed EGF-induced Gab1-Shp2 interaction in HEK293T cells treated with Fumos using immunoprecipitation. In the absence of EGF stimulation, Gab1 were not detected in Shp2 immunoprecipitates (Fig. 3a). Shp2 was co-immunoprecipitated with increased tyrosine-phosphorylated Gab1 (pY-Gab1) in EGF-stimulated cells, and much less Gab1 proteins were found after Fumos treatment in the presence of EGF stimulation. Inhibition of EGF-stimulated Gab1-Shp2 association by Fumos was confirmed by reciprocal co-immunoprecipitation (Fig. 3a,b). Treatment of cells with Fumos alone increased the tyrosine-phosphorylation of Gab1. In previous report, inhibition of Shp2 resulted in an increase in the tyrosine phosphorylation of Gab1, albeit to different extents^38^. Our results are consistent with these previous studies. We also analyzed EGF-induced Gab1-Shp2 interaction treated with Fumos in MDA-MB-231 cells, and got the similar results as in HEK293T cells (Supplementary Fig. S1 online). All data indicate Fumos indeed inhibits the Gab1-Shp2 association after EGF simulation. Surprisely, Fumos alone induces Gab1-Shp2 association in both cell lines. The possible reason is that Fumos may induce the cellular stress leading to the interaction between Gab1 and Shp2.

RasGAP functions as a negative regulator of Ras and binds to tyrosine-phosphorylated Gab1^39^,^40^.Shp2 could dephosphorylate RasGAP binding sites to disengage RasGAP from Gab1 and sustain Ras activation. After Fumos treatment, RasGAP coimmunoprecipitated with Gab1 was dramatically increased in EGF-stimulated cells leading to the inhibition of Ras activation (Fig. 3b,d).

### Fumos doesn’t inhibit EGF-induced AKT activation

**Figure 4:**
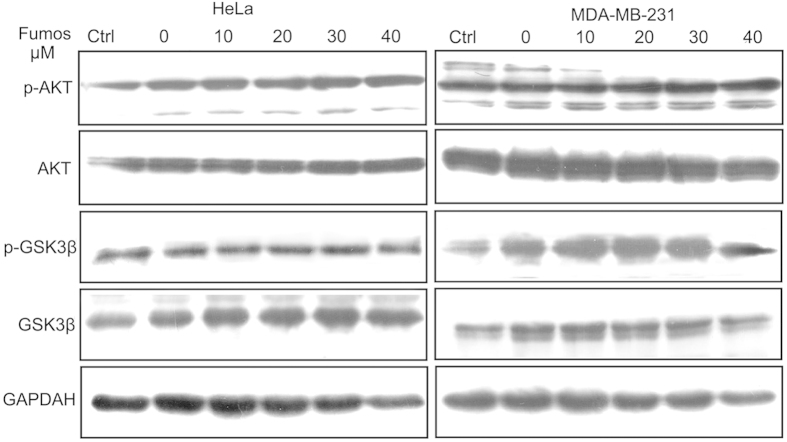
Fumos doesn’t inhibit EGF-induced AKT activation. Fumos doesn’t inhibit EGF-induced AKT activation. Serum starved cells were pretreated with various concentrations of Fumos (0, 10, 20, 30, 40 μM) for 24 h and then stimulated with EGF (20 ng/ml, 5 min). DMSO treatment as control. Western blotting analysis for p-AKT, AKT, p-GSK3β and GSK3β.

AKT is downstream of PI3K and linked to glucose metabolism, apoptosis, cell proliferation, transcription and cell migration^41^,^42^. Phospho-AKT (Ser473) is a hallmark for PI3K activity in intact cells^43^. The tyrosine phosphatase Shp2 is required for activation of phosphatidylinositol 3-kinase/AKT by growth factors independent of its PTP activity^44^. Our results showed that Fumos did not inhibit the phosphorylation of AKT and its substrate GSK3β (Fig. 4a).

### Fumos inhibits tumor cell invasion

**Figure 5:**
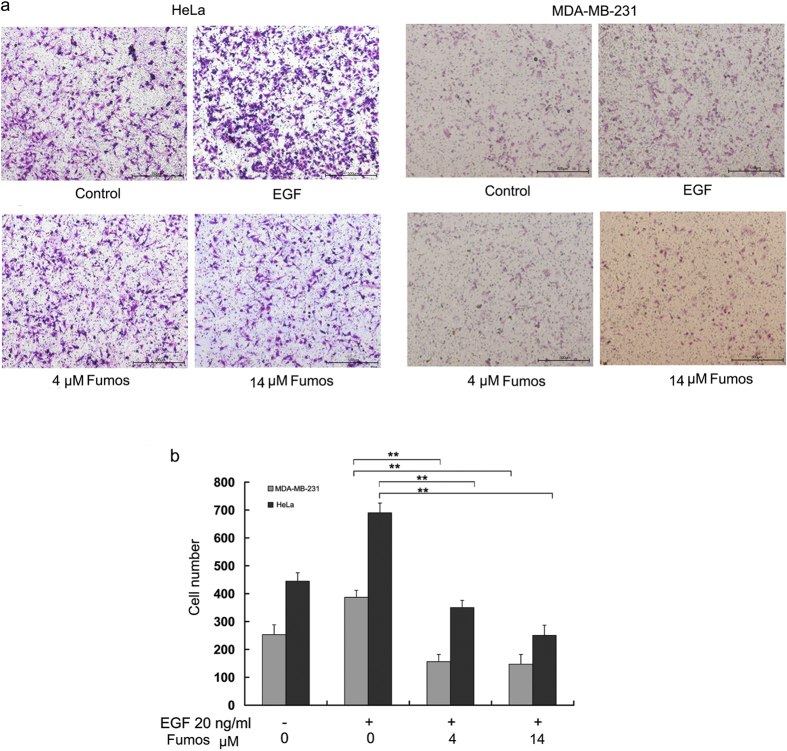
Fumos inhibits EGF-induced cell invasion. (**a**) The cells were treated at indicated concentrations of Fumos (4 μM and 14 μM) for 24 h. Fumos inhibited cell invasion as determined by Transwell assay in MDA-MB-231 cells and HeLa cells. The migratory cells were detected by crystal violet staining and photographed. DMSO treatment as control. The scale bar is 500 μm. (**b**) The histogram of cell invasion analysis. Cells were randomly selected and counted for ten scopes of microscope. Values are mean ± S.E.M. of three experiments. HeLa **p = 0.001 < 0.01vs EGF (F = 1725); MDA-MB-231 **p = 0.001 < 0.01vs EGF (F = 658), one-way ANOVA followed by Tukey’s test.

Shp2 regulates cell migration, chemotaxis and invasion by activating several Src-family kinases in metastatic triple-negative breast cancer (TNBC) cells^45^. To examine the effect of Fumos on the invasion of two human cancer cell lines (HeLa and MDA-MB-231), Transwell assay was used to characterize cell invasion in the pressence of 0, 4 and 14 μM Fumos. Cells were placed on the upper layer of a cell permeable membrane in Transwell chamber. After incubating with Fumos, the cells that have migrated through the membrane were stained and counted. As shown in Fig. 5, Fumos significantly inhibited the invasion of both tumor cell lines after incubation for 24 h. Moreover, Fumos inhibited tumor invasion in a dose-dependent manner. The inhibition of cell invason was not due to the cytotoxicity of Fumos, because we observed no significant difference in cell viability at these concentrations.

### Fumos affects the phosphorylation of Src involved in cell invasion

**Figure 6:**
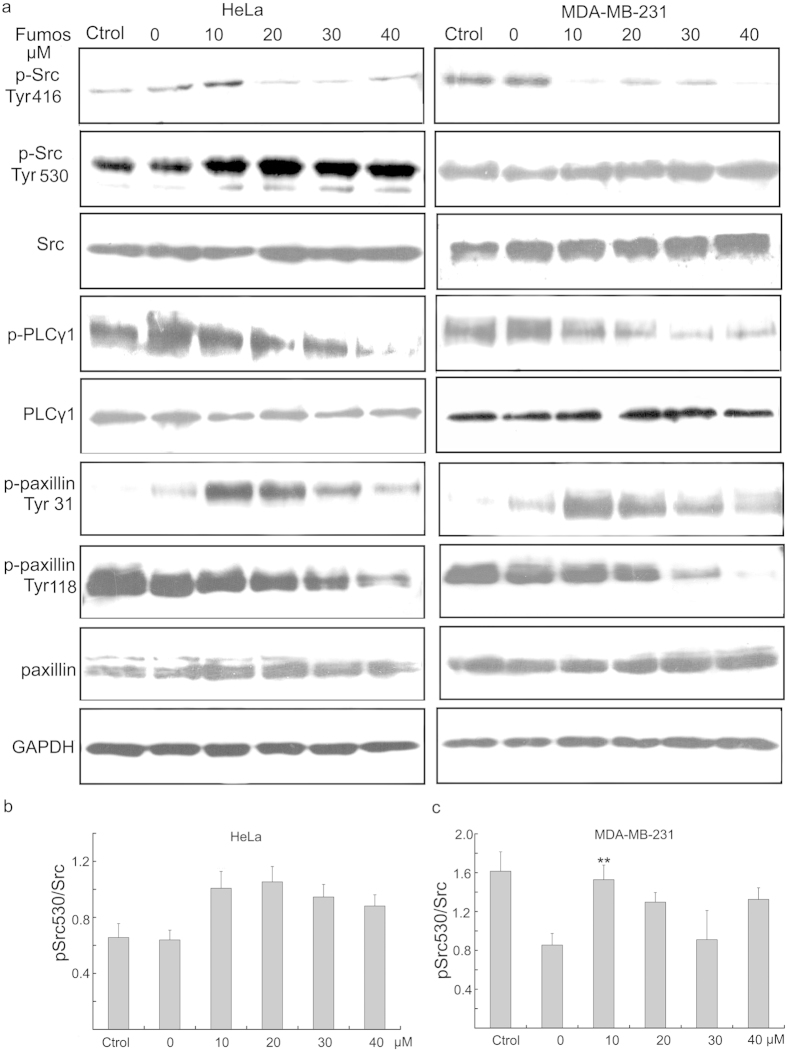
Fumos affects the phosphorylation of Src and Paxillin. (**a**) Serum starved cells were pretreated with various concentrations of Fumos(0, 10, 20, 30, 40 μM) for 24 h and then stimulated with EGF (20 ng/ml, 5 min). DMSO treatment as control. Western blotting analysis for the expression of p-Src(Tyr416) p-Src (Tyr530), Src, p-PLCγ1, PLCγ1, p-Paxillin(Tyr118), p-Paxillin (Tyr31) and Paxillin. (**b,c**) Densitometric analysis of pSrc530/Src in the Western blots of Hela and MDA-MB-231, respectively. Bars represent mean ± S.E.M. values from three experiments, **p = 0.006 < 0.01/F = 8 vs EGF, one-way ANOVA followed by Tukey’s test.

Next, we investigated whether Fumos was able to inhibit the activation of Src. Src is a non-receptor cytoplasmic tyrosine kinase which overexpresses in many tumors and plays an important role in promoting tumor invasion and migration^46^. Sausgruber *et al.* found a decreased phosphorylation of the activating Src at Tyr416 and phosphorylation of Paxillin at Tyr118 and Tyr31 upon depletion of Shp2^45^. Exposure of MAD-MB-231 cells and HeLa cells to Fumos resulted in a dose-dependent downregulation of Src Tyr416 phosphorylation (Fig. 6), a hallmark of Src activation^47^. Moreover, the phosphorylation of PLCγ1, a substrate of Src, was also decreased after Fumos treatment, indicating the attenuation of Src activity (Fig. 6). The inhibitory phosphorylation of Tyr 530 acts as a major negative regulator of Src^47^. The phosphorylated Tyr 530 of Src moderately increased after Fumos treatment (Fig. 6b,c). Paxillin is involved in the progress of cell adhesion, spreading, and motility^48^,^49^. After Fumos treatment, the phosphorylation of Paxillin at Tyr31 peaked at low concentrations of Fumos and reduced at high concentrations in both cell lines. The phosphorylation of Paxillin at Tyr118 decreased with the increasing concentrations. All data suggested Fumos affects the phosphorylation of Src and Paxillin which are involved in cell invasion.

## Discussion

Shp2 is a non-receptor phosphatase and has been considered as a proto-oncogenic protein^50^. Although Shp2 is proposed as a cancer drug target for many years, few Shp2 inhibitors were reported in clincal study. The main barrier in the discovery of PTP inhibitors is the selectivity. Because the highly conserved active site with Shp1 and PTP1B, achieving selective Shp2 inhibition is a challenge^19^. Non-selective inhibitors of Shp2 may induce adverse effects. For example, Shp1 negatively regulates multiple signaling pathways in a number of hematopoietic lineages, loss of Shp1 function leads to severe autoimmune and immunodeficiency syndrome^51^. Fumos showes higher selectivity against other phosphatases and higher activity than sodium orthovanadate, a general inhibitor for PTPs. Fumos exhibits approximately 2-, 5-, and 6-fold selectivity for Shp2 over PTP1B, TCPTP and Shp1, respectively, whose structures are closely related to Shp2 (Table 1). Fumos showes very little inhibition toward other PTPs such as HePTP, Lyp, STEP, PTPH1, PTPRA, Cdc25b and MEG2. SPR analysis revealed that Fumos directly binds to Shp2 with K_D_ = 40 nM. In addition, Lineweaver-Burk analysis suggested that Fumos is a classical non-competitive inhibitor of Shp2. Futhermore, molecular docking between Fumos and Shp2 explains the specificity of Fumos for Shp2. Based on the docking results, Fumos is predicted to bind to PTP loop, WPD loop and other sites. The docking data showed that Fumos did not totally ocupy in the active site, and partially explain the fact that Fumos is a non-competitive inhibitor of Shp2.

The N-SH2 domain of Shp2 has been reported to bind to the catalytic domain resulting in autoinhibition of the Shp2 PTP activity^52^. Full-length Shp2 is 4-fold less sensitive to Fumos than the Shp2 PTP domain, indicating that the N-SH2 domain of Shp2 interferes with the interaction between Fumos and Shp2, and Fumos preferentially inhibits activated Shp2.

**Figure 7:**
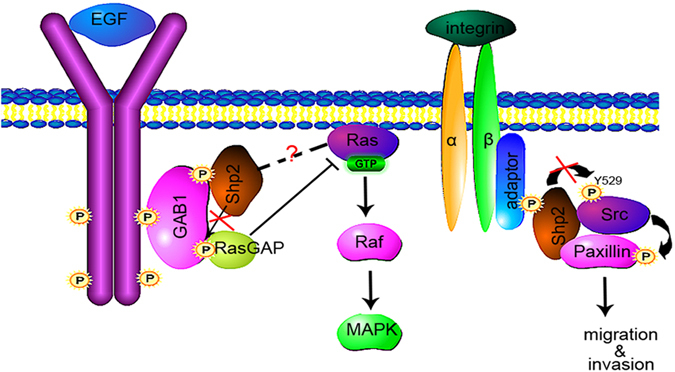
Scheme of the proposed mechanism of action of Fumos. EGF induces the activation of EGFR. Subsequently, Gab1 is associated with the activated EGFR, and Shp2 can dephosphorylate Gab1 on RasGAP binding sites disengaging RasGAP from Ras activation^39^. The PTP activity of Shp2 is required for full activation of Ras. Fumos inhibits the Gab1-Shp2 interaction leading to downregulate the acitivation of Ras-ERK. Shp2 also induces the activation of Src by increasing autophosphorylation at Tyr416 and dephosphorylating the inhibitory site Tyr530, which leading to the phosphorylation of paxillin^45^. Fumos inhibits Src activation by downregulating the phosphorylation of Src and paxillin. The red X symbol represents the effective site of Fumos.

When Shp2 is activated by the receptor of the tyrosine kinase signal such as epidermal growth factor, insulin, and insulin-like growth factor-1 receptor, the PTP domain is released from autoinhibition leading to exposure of the PTP domain and catalytic activation^5^,^53^. Shp2 via its SH2 domains binds to tyrosinephosphorylated docking proteins such as Grab2, Shc and Gab1, and subsequently promotes Ras activation. The active form of Ras in turn activates the Raf-MEK-ERK cascade, which stimulates cellular processes including cell proliferation, differentiation, or survival (Fig. 7). The precise mechanism by which Shp2 promotes Ras activation remains controversial. Three principal models have been proposed^4^,^6^. One modle is that once Gab1 is associated to the activated EGFR, Shp2 can dephosphorylate the binding sites of Gab1 to disengage RasGAP leading to Ras activation^27^. Activated Shp2 binds to the tyrosine-phosphorylated docking protein Gab1 through its SH2 domain^37^. Fumos blocks EGF-induced Gab1-Shp2 association and increases the interaction between RasGAP and Gab1 resulting in decreased Ras activation. Therefore, Fumos inhibits EGF-induced activation of ERK1/2 downstream of Ras in cells expressing wild type Ras. Suprisely, Fumos treatment alone leads to Shp2-Gab1 interaction in both cell lines. In previous reports, the Gab1-Shp2 interaction could be induced not only by EGF, but also by oxidative stress or partial hepatectomy^40^,^54^. Gab1 is required for H_2_O_2_-induced c-Jun N-terminal kinase (JNK) activation dependent on Shp2 in oxidative stress^54^. Fumos alone may induce the cellular stress leading to the interaction between Gab1 and Shp2. After EGF stimulation, Fumos inhibited Gab1-Shp2 association due to the inhibition of Shp2. We also couldn’t exclude the possibility that other proteins help to dissociate Shp2 from Gab1 during EGF stimulation after Shp2 inhibition.

In MDA-MB-231 cells expressing mutant Ras, Fumos interrupts EGF-induced Gab1-Shp2 association, but it could not inhibit the activation of Erk1/2 downstream of mutant Ras (Supplementary Fig. S1 online). Fumos also shows little effects on the Erk1/2 activation induced by PMA in a Ras-independent manner. In conclusion, Fumos has no effect on the Shp2-independent activation of Erk1/2 induced by PMA or oncogenic Ras.

Fumos also shows no effects on the activation of the PI3K/AKT pathway, which is Shp2-independent. In addition, we tested whether enhanced expression of Shp2 or PTP1B could counteract the inhibitory effect of Fumos. HEK293T cells with transfection of Shp2 partially recovered the ERK activity after Fumos treatment. But cells with transfection of PTP1B showed little recovery (Supplementary Fig. S2 online). These are indirect proofs for the inhibition of Shp2 by Fumos.

Fumos also down-regulates Shp2-Src signaling pathway and efficiently prevents tumor cell invasion. Src is an Shp2 effector involved in EGF-stimulated cell migration and invasion^49^. Src as a key molecule in tumor progression can provide oncogenic signals for cell survival, invasion and metastasis by phosphorylating tyrosine residues on substrates such as the focal adhesion kinase (FAK), Crk-associated substrate, Paxillin, downstream of RTKs and integrins^55^. Src is regulated by phosphorylation at key sites, including the autophosphorylation site Tyr416 and the inhibitory site Tyr530. Previous studies have been reported that Shp2 releases the inhibition of Src indirectly by separating the Src-inhibiting kinase(Csk) from the paxillin-Src complex^47^,^49^. But Sausgruber *et al.* demonstrated that Shp2 interacts directly with Tyr530 of Src and enhances the dephosphorylation at Tyr530 leading to the activation of Src(Fig. 7). They also found a decrease of phosphorylation of the activating Src at Tyr416 upon depletion of Shp2^45^. Fumos, as a selective inhibitor of Shp2, could significantly reduce the phosphorylated Tyr416 but only slightly increase the phosphorylated Tyr530 of Src. This result may be due to lower sensitivity of phospho-specific antibody to detect Tyr530 phosphorylation^47^. Sausgruber *et al.* also found that depletion of Shp2 decreased phosphorylation of Paxillin at Tyr118 and Tyr31^45^.The phosphorylation of Paxillin at Tyr31 and Tyr118 was reduced with the increased concentrations of Fumos. Fumos may prevent tumor cell invasion by down-regulating Src signaling pathway.

In this study, we discovered Fumos as a novel selective inhibitor of Shp2. Our results suggest that Fumos presents specific inhibition of Shp2-dependent signaling and is worthy of further research as a potential compound for the treatment of Shp2-related diseases.

## Methods

### Materials

Fumos was isolated and purified according to the methods described by research group of Prof. Duqiang Luo^28^,^29^. p-ERK, ERK, p-AKT, p-GSK3β, paxillin and GSK3β antibodies were purchased from Cell Signaling Technology. Antibodies p-Tyr, p-paxillin, p-Src419, p-Src529, PLCγ1 and p-PLCγ1 were purchased from Signalway Antibody LLC, SAB. Gab1 and Myc-tag antibodies were purchased from Abcam and Santa Cruz Biotechnology respectively.

### Cell culture

HEK293T, MDA-MB-231and HeLa cell lines were obtained from CRC/PUMC. Cell lines were maintained in Dulbecco’s modified Eagle’s medium supplemented with 10% fetal bovine serum. Transfections were performed in 6-well plates according to manufacturer (Invitrogen Lipofectamine LTX).Briefly, for transient transfection of HEK293T cells, subconfluent cells in 6-well plates were incubated with 2 ml of Dulbecco’s modified Eagle’s medium containing 2.5 μg of total DNA and 7.5 μl of each Lipofectamine and Plus reagent (Invitrogen) and then serum-starved in DMEM/0.1% bovine serum albumin for 18 h. After Fumos treatment for 24 h, cells were stimulated for 5 min with 20 ng/ml EGF.

### Computer Docking

Computer docking was performed by software Discovery Studio(DS2.5). Fumos was taken energy optimization using the DS minimize ligands module. Computer docking was performed using the X-ray crystal structure of human Shp2 (PDB ID: 3MOW)^56^. The N-SH2 domain of Shp2 was removed from the 3D structure before the computer docking analysis. PDB struture was pretreated using Clean protein and Prepare protein module. The optimal molecular docking was obtained by using the Libdock module of DS.

### Recombinant PTP Proteins

Plasmids for expression of His-tag fusion proteins of human PTP were constructed in PET-28a by PCR subcloning techniques. The constructions contain only the PTP domain except Full-length Shp2. Full-length Shp2 containing Tyr279-to-Ala mutant was generated by PCR-based mutagenesis. All constructs were verified by DNA sequencing. PTP-His fusion proteins were expressed in *Escherichia coli* BL21 and lysed in the buffer (50 mM NaH_2_PO_4_, 300 mM NaCl, 250 mM imidazole, 1 mM dithiothreitol plus 5% glycerol), purified with Ni-NTA Magnetic Agarose Beads (Qiagen). The His-tag recombinant purification protocol was carried out according to the purification under native conditions of the QIAexpress System.

### PTP assay

The assay was set in wells of 96-well plate with a final volume of 100 μl of reaction mixture containing 10 mM of NaAc-HAc, 1 mM of ethylene diamine tetraacetic acid, 1 mM of DL-dithiothreitol and 2% of dimethyl sulfoxide, pH 5.5. Activity of the enzyme was assayed by incubating recombinant PTP protein or mutant PTP with various concentrations of Fumos in the mixture at 37 °C for 30 min. Absorbance at 405 nm was detected immediately after addition of varied concentrations of substrate, *p*-nitrophenyl phosphate (*p*NPP) to each corresponding well.

### Irreversibility assessments for Fumos

Recombinant Shp2 was mixed with the inhibitor at a final concentration of 100-fold IC_50_ or with vehicle control (DMSO) respectively. Sodium orthovanadate was used as a positive control compound. After incubation at room temperature for 30 min, the Enzyme-inhibitor was diluted to one-fold and mixed with 40-fold pNPP (5 M) for enzymatic activity.

### Surface Plasmon Resonance Analysis

One small molecule (10 mM) was spotted on the 3D sensor chip. Positive and negative controls on the chip were monitored for binding kinetics to verify immobilization. All experiments were performed using Plexera’s Plex Array SPRi instrumentation, visualized using Instrument Control software (ICS) and analyzed using Plexera Data Explorer software.

Proteins were injected in the following order: Shp2 full-length protein (12 nM, 36 nM, 108 nM, 324 nM), Shp2 Y279A protein (35 nM, 70 nM,140 nM, 280 nM, 560 nM). Each protein was injected at a flow rate of 1 μl/sec. The association duration was 200 sec and dissociation duration was 300 sec. Regeneration between injections was performed with 600 μl of 10 mM glycine-HCl, pH = 2.0. The data were analyzed by Plexera Data Explorer software.

### Ras Activation Assay

Active Ras was detected by use of the RBD of raf-1 fused to glutathione S-transferase (GST) as described previously^57^. Cells were lysed in MLB buffer (25 mM Hepes, 150 mM NaCl, 1% Nonidet P-40, 0.25% sodium deoxycholate, 10% glycerol, 25 mM NaF, 1 mM Na_3_VO_4_, 10 mM MgCl_2_, 1 mM EDTA, pH 7.5) supplemented with complete EDTA-free protease inhibitor mixture^58^. Total 500 μg cell lysate was incubated with 10–20 μg of GST-RBD bound beads(Merck) overnight at 4 °C. The beads were pelleted by centrifugation and washed three times with MLB buffer. The beads were boiled in SDS loading buffer and subjected to electrophoresis, followed by immunoblotting with an anti-Ras antibody (Santa Cruz Biotechnology).

### Transwell assay

The invasion assay was performed in Transwell chambers according to Corning methods. Briefly, the upper chamber of a Transwell insert (8 mm pore size) was coated with 40 μl 1:6 mixture of Matrigel (BD Biosciences, Bedford): Opti-MEM (Invitrogen) for MDA-MB-231 and 40 μl 1:12 mixture of Matrigel: Opti-MEM (Invitrogen) for HeLa cells and dried for 2 h at 37 °C. The lower chamber was filled with serum-free media containing 10 ng/ml EGF. Cells were seeded into upper chamber at a density 5 × 10^4^ cells/ml per well in serum-free media. Cells were treated with series concentrations of Fumos for 24 h. The top aspect of the membrane was gently swabbed the inside of each insert using cotton swabs, and cells that invaded on the lower surface of the membrane were fixed using methanol and stained with crystal violet (Beyotime Institute of Biotechnology). Random fields were counted and photographed by microscope (Olympus).

### Immunoprecipitations

Cells were washed with cold PBS twice and lysed in cell lysis buffer for 10 min on ice. Then the lytic cells were centrifugated with 13,000 g for 10 min at 4 °C. The supernatant was transferred to a fresh conical centrifuge tube on ice and added 1 μg of the appropriate control IgG, together with 20 μl of resuspended volume of Protein A/G PLUS-Agarose (Santa Cruz Biotechnology, CA), then incubated at 4 °C for 30 min. The pellet beads were centrifugated at 1,000 g for 5 min at 4 °C. The supernatant with approximately 100–500 μg total cellular protein was incubated with 2 μg primary antibody against Shp2 or Gab1 for 7 h at 4 °C. Then lysates containing protein-antibody complexes were incubated with 20 μl of Protein A/G PLUS-Agarose at 4 °C on a rotating device overnight. Immunoprecipitates were collected by centrifugation at 2,500 rpm and washed for 4 times with 1 ml lysis buffer, then suspended in the loading buffer, boiled, and analyzed by SDS-PAGE.

### Western Blot

Cells were seeded in a culture bottle at 37 °C, under 5% CO_2_ and 95% air. The cells were serum-starved for 18 h, treated with Fumos (0 μM, 10 μM, 20 μM, 30 μM and 40 μM for 24 h) and then stimulated with EGF (20 ng/ml, 5 min). Then cells were collected and dissolved in the lysis buffer (50 mM Hepes-NaOH, 100 mM NaCl, 0.5%NP-40, 2.5 mM EDTA, 10% glycerol, 1 mM DTT, 1 mM PMSF, 0.7 μl/ml pepstatin, 0.5 μg/ml leupetin, 2 μg/ml aprotinin). Cell lysates were centrifuged at 4 °C with the speed of 10,000 g for 15 min. After centrifugation, the supernatant was collected. 30 μg proteins were loaded on SDS-PAGE. After electrophoresis, proteins were transferred onto PVDF meber, and immunobloted with specific antibodies, and visualized using ECL reagent. Each membrane was stripped and reprobed with anti-GAPDH antibody to ensure equal protein loading. The bands were densitometrically scanned and analyzed using a BIO-RAD Gel Analysis System.

### Statistics

SPSS software was used for statistical analysis. Statistical significance of differences was assessed using one-way ANOVA followed by a Tukey’s post hoc test for multiple comparisons. Differences between groups were considered statistically significant at values of *p* < 0.01.

## Additional Information

**How to cite this article**: Chen, C. *et al.* Discovery of a Novel Inhibitor of the Protein Tyrosine Phosphatase Shp2. *Sci. Rep.* **5**, 17626; doi: 10.1038/srep17626 (2015).

## Supplementary Material

Supplementary Information

## Figures and Tables

**Table 1:** Inhibition of PTPs by Fumos *in vitro*.

PTP	IC_50_ (μM)	IC_50_ selective
Shp2	6.31 ± 0.52	1
PTP1B	14.04 ± 0.60	2
TCPTP	29.65 ± 0.42	5
SHP1	34.70 ± 0.37	6
HePTP	>100	>16
Lyp	>100	>16
STEP	>100	>16
PTPH1	>100	>16
PTPRA	>100	>16
Cdc25b	>100	>16
Meg2	>100	>16
Shp2 FL	26.52 ± 0.37	4
Shp2 Y279A	–	–

Each experiment was performed in triplicate. Data are presented as mean ± S.E.M. All PTP proteins contain only PTP domain except full lenth Shp2 (Shp2 FL) and Shp2 Y279A. Shp2 Y279A shows no enzyme activity. Na_3_VO_4_ potently inhibited Shp2 PTP domain and Shp2 FL with an IC_50_ of 620 μM and 630 μM, respectively.
