# Supplementary material for: Discovery of a Novel Inhibitor of the Protein Tyrosine Phosphatase Shp2
Source: Sci Rep. 2015 Dec 2;5:17626. doi: 10.1038/srep17626 (PMC4667271; doi:10.1038/srep17626)
Supplement: Supplementary Information [file srep17626-s1.pdf]

Discovery of a Novel Inhibitor of the Protein Tyrosine Phosphatase Shp2

Chuan Chen[#], Mengmeng Cao[#], Siyu Zhu, Cuicui Wang, Fan Liang, Leilei Yan, Duqiang Luo[*]

[a]*College of Life Science, Key Laboratory of Medicinal Chemistry and Molecular Diagnosis of the Ministry of Education, Hebei University, Baoding, Hebei 071002, P.R. China*

Correspondence authors: Professor Du-Qiang Luo, Tel: 86-0312-5079364
E-mail: duqiangluo@163.com College of Life Science, Key Laboratory of Medicinal Chemistry and Molecular Diagnosis of the Ministry of Education, Hebei University, Baoding, Hebei 071002, P.R. China.

Supplementary Figure

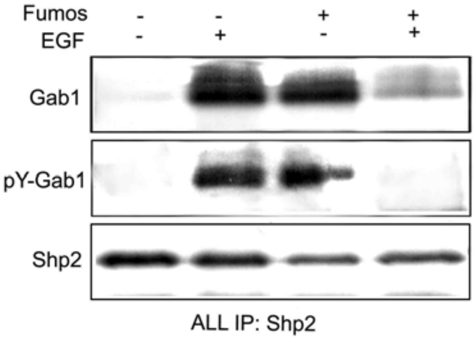

Figure S1. Fumos inhibits Gab1-Shp2 association in MDA-MB-231 cells.

MDA-MB-231 cells were serum-starved for 18 h, preincubated with or without Fumos (20 μM 24 h), and then treated with EGF (20 ng/ml, 5 min). Shp2 was immunoprecipitated from cell lysate supernatants. Immunoprecipitates were analyzed by immunoblotting with antibodies to Gab1, pY and Shp2.

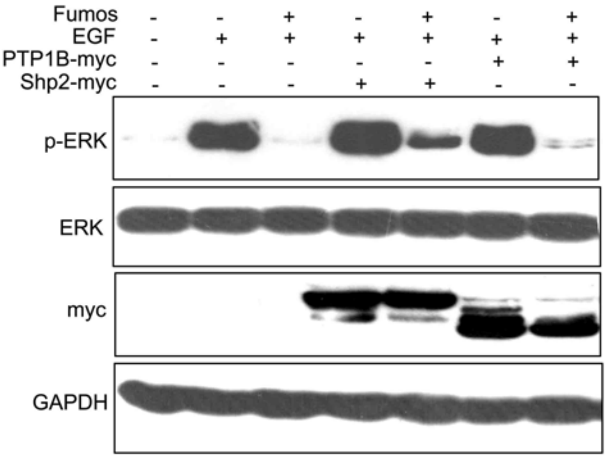

Figure S2. Fumos inhibits Shp2-mediated ERK1/2 activation in human cells.

HEK293T cells were transiently transfected with a pCMV empty vector, expression vectors for PTP1B and Shp2, and either untreated or treated with 20 μM Fumos for 24 h, respectively. Total cell lysates were immunoblotted with antibodies specific for myc-tag, p-ERK1/2 and ERK1/2.
